# Survival of *Escherichia coli* O157:H7 during Moderate Temperature Dehydration of Plant-Based Foods

**DOI:** 10.3390/foods10092162

**Published:** 2021-09-13

**Authors:** Yadwinder Singh Rana, Philip M. Eberly, Quincy J. Suehr, Ian M. Hildebrandt, Bradley P. Marks, Abigail B. Snyder

**Affiliations:** 1 Department of Food Science, Cornell University, Ithaca, NY 14850, USA; yr246@cornell.edu; 2 Department of Food Science, The Ohio State University, Columbus, OH 43210, USA; eberly.82@buckeyemail.osu.edu; 3 Department of Biosystems and Agricultural Engineering, Michigan State University, East Lansing, MI 48824, USA; suehrqui@msu.edu (Q.J.S.); hildeb44@msu.edu (I.M.H.); marksbp@msu.edu (B.P.M.)

**Keywords:** plant-based, low-moisture food, process validation

## Abstract

The effect of moderate-temperature (≤60 °C) dehydration of plant-based foods on pathogen inactivation is unknown. Here, we model the reduction of *E. coli* O157:H7 as a function of product-matrix, *a_w_*, and temperature under isothermal conditions. Apple, kale, and tofu were each adjusted to *a_w_* 0.90, 0.95, or 0.99 and inoculated with an *E. coli* O157:H7 cocktail, followed by isothermal treatment at 49, 54.5, or 60.0 °C. The decimal reduction time, or *D*-value, is the time required at a given temperature to achieve a 1 log reduction in the target microorganism. Modified Bigelow-type models were developed to determine *D*-values which varied by product type and *a_w_* level, ranging from 3.0–6.7, 19.3–55.3, and 45.9–257.4 min. The relative impact of *a_w_* was product dependent and appeared to have a non-linear impact on *D*-values. The root mean squared errors of the isothermal-based models ranged from 0.75 to 1.54 log CFU/g. Second, we performed dynamic drying experiments. While the isothermal results suggested significant microbial inactivation might be achieved, the dehydrator studies showed that the combination of low product temperature and decreasing *a_w_* in the pilot-scale system provided minimal inactivation. Pilot-scale drying at 60 °C only achieved reductions of 3.1 ± 0.8 log in kale and 0.67 ± 0.66 log in apple after 8 h, and 0.69 ± 0.67 log in tofu after 24 h. This illustrates the potential limitations of dehydration at ≤60 °C as a microbial kill step.

## 1. Introduction

Dehydration has served as a preservation process for hundreds of years wherein microbial growth is inhibited through the removal of available water from a food product [1]. Typical commercial dehydration is a mild thermal process often ranging from 40 to 80 °C with forced air circulation [2,3,4]. The kinetics of thermal pathogen inactivation under complex dehydration conditions is poorly understood. Previous fruit dehydration research has primarily evaluated microbial inactivation at temperatures >60 °C [5,6,7]. However, processors, especially small-scale processors, may use temperatures ≤60 °C for dehydration of plant-based foods including dried fruits, kale chips, kale smoothie powders, spices and herbs, and vegan jerky (dehydrated tofu or mushrooms). Fresh produce has been associated with outbreaks of foodborne illness attributed to a variety of pathogens including diarrheagenic *E. coli* [8,9], although there are limited reports of foodborne disease outbreaks attributed to dehydrated plant-based food products [10,11]. However, the relative efficacy of moderate-temperature (≤60 °C) dehydration of plant-based foods on pathogen inactivation remains unknown.

Heat-assisted dehydration involves dynamic temperature and moisture levels, which increases the complexity of validating microbial inactivation outcomes. Accurate assessments of microbial inactivation must account for these dynamics. Processing variables (air currents, temperature, and humidity) within the dehydration unit affect product temperature and water activity (*a_w_*), critical factors for pathogen inactivation. Processing variables are often unit-specific and dependent on equipment design features [12]. In addition, factors dependent on food-type (initial moisture content, moisture diffusivity, thickness) and dehydrator operation (fill density, tray position, and initial product temperature) can also affect microbial kill [3,12,13]. Uneven humidity and thermal distribution may result in “cold spots”, locations of lower temperature and higher *a_w_* relative to the rest of the unit [13]. In thermal processes at consistent moisture levels, the cold spot represents the location of least kill. However, because of the higher *a_w_* at these locations relative to “hotter spots”, the cold spot may not represent the worst-case scenario location for microbial inactivation. In these cases, the driest spot, even if it achieves a higher treatment temperature, may represent the worst-case scenario for microbial lethality during dehydration. Additionally, moderate temperatures (≤60 °C) and extended come-up times may create suitable conditions for microbial growth [14,15]. Temperatures between 4.4 °C to 60 °C (40–140 °F), sometimes referred to as the “temperature danger zone,” and *a_w_* above 0.95 support the growth of many spoilage and pathogenic bacteria and can potentially lead to toxin production from such foodborne pathogens as *Staphylococcus aureus* and *Bacillus cereus* [15,16]. Given that the *a_w_* of plant-based products is initially >0.95 prior to dehydration, these conditions may support microbial growth until sufficient moisture has been removed.

In the U.S., the Preventive Controls for Human Food Rule of the Food Safety and Modernization Act (FSMA) established a regulatory basis for validation of process preventive controls, which could include thermal dehydration [17]. While larger businesses are more likely to have validated their processes, and more likely use higher temperatures, smaller food businesses are often engaged in operations that may have less empirical evidence supporting efficacy. Recent studies reporting microbial thermal inactivation models in low *a_w_* food systems include potato discs, ground almonds, and whey powder [18,19,20]. However, those systems are appreciably different than heat-assisted dehydration of plant-based foods and the application of existing models to additional legacy processes must be explored to ensure microbial safety in other low *a_w_* foods. The objective of this study was to evaluate *Escherichia coli* O157:H7 inactivation kinetics in isothermal and iso-moisture (40–60 °C, 0.90–0.99 *a_w_*) experiments and in pilot-scale dehydrator (60 °C) trials on plant-based foods to assess the feasibility of using 60 °C dehydration as a thermal kill step.

## 2. Materials and Methods

### 2.1. Food Samples

Three plant-based products, extra firm tofu (Nasoya foods, Ayer, MA, USA), fuji apples (Archer farms, Minneapolis, MN, USA), and fresh-cut kale (Taylor farms, Salinas, CA, USA) were purchased from a national retailer. The samples were stored under refrigeration conditions (~4 °C) up to 24 h before use. Initial background microbiota counts in the samples were determined by plating uninoculated product samples onto tryptic soy agar (TSA, BD, Thermo Fisher Scientific, Waltham, MA, USA), followed by incubation at 35 ± 2 °C for 24 ± 3 h.

### 2.2. Inoculum Selection and Preparation

*E. coli* O157:H7 32C, *E. coli* O157:H7 32DB [21], *E. coli* O157:H7 meat-1, and *E. coli* O157:H7 meat-2 provided from the culture collection of Dr. Ahmed Yousef (Department of Food Science and Technology, The Ohio State University, Columbus, OH, USA) were used to make a four-strain cocktail for this study. Cultures were maintained at −80 °C in 20% glycerol stock. A loopful of frozen stock was initially inoculated into tryptic soy broth (TSB, BD, Thermo Fisher Scientific, Waltham, MA, USA) and incubated at 35 ± 2 °C for 24 ± 3 h. Broth suspension was streaked onto TSA plates and incubated at 35 ± 2 °C for 24 ± 3 h. An isolated colony was transferred from stock plates into TSB followed by incubation at 35 ± 2 °C for 20 ± 3 h. After incubation the culture broth was centrifuged at 2817× *g* for 5 min and 4 °C (Eppendorf, NY, USA), the cell pellet was washed and resuspended in 0.1% peptone water (PW) (BD, Thermo Fisher Scientific, Waltham, MA, USA). This process was repeated for each of the four *E. coli* strains and prior to inoculation, equal volumes of each bacterial strain were mixed together to make a cell cocktail. The initial cell count was determined to be ~10^8^ CFU/mL by plating cell dilutions onto TSA.

### 2.3. Sample Inoculation

An hour before inoculation, food samples were taken out of refrigeration (~4 °C) and allowed to equilibrate to room temperature (~20 °C). Initial product *a_w_* was recorded using Aqualab 4-TE (METER group, Pullman, WA, USA). The *a_w_* of apple and tofu slices, as well as chopped kale was adjusted for isothermal experiments using a dehydration unit (Weston pro-2400, Weston, Southern Pines, NC, USA) operated at 60 °C for up to 2.5 ± 0.5 h (kale), 8 ± 2 h (apple) and 20 ± 2 h (tofu) to achieve the target *a_w_* level (0.99 ± 0.01, 0.95 ± 0.01, or 0.90 ± 0.01). Inoculum (1 mL) was concentrated by centrifugation (2817× *g* for 5 min at 4 °C) and resuspended in 10 µL PW to minimize the food sample *a_w_* change by reducing the volume of inoculum applied. For sample preparation, 5 g of *a_w_* adjusted sample was added to a sterile Whirl-Pack^®^ bags (Nasco, Fort Atkinson, Madison, WI, USA) followed by inoculation with 10 µL concentrated cell cocktail (~10^8^ CFU/g). Inoculum was distributed by hand massaging and the final *a_w_* was confirmed (±0.02 of the target *a_w_*) before isothermal treatment.

### 2.4. Isothermal Studies

Samples were heat-sealed and treated at 49 °C, 54.5 °C, and 60.0 °C (120, 130, and 140 °F) for up to 300, 180, and 30 min, respectively, in a water bath (FSGPD05, Thermo Fisher Scientific, Waltham, MA, USA). Come-up-time was initially verified for two samples of each product using a thin wire K-type thermocouple (5SRTC-GG-K-24-36, Omega Engineering, Norwalk, CT, USA) inserted in the center of the bag. The isothermal time series was initiated when the sample temperature was within 0.5 °C of the set temperature, with the come-up-time defined as the heating time needed to reach this temperature. Samples were pulled after predetermined time intervals between 5 and 60 min, depending on temperature, and transferred to an ice bath for ~3 min to halt the thermal treatment. PW (0.1% *w*/*v*, 5 mL) was added to the bags and the samples were hand stomached for ~1 min and appropriate dilutions plated onto TSA. Plates were incubated for 24 ± 3 h at 35 ± 2 °C. Three biological replications were performed for each experimental condition.

### 2.5. Inoculated Pack Dehydration Studies

**Figure 1:**
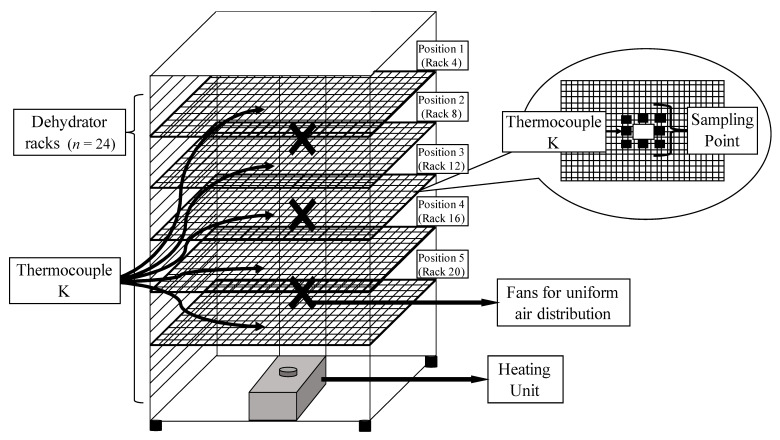
Schematic of the dehydration unit with five sampling rack positions ranging from top (rack 4) to the bottom (rack 20) of dehydrator (Total racks in the unit = 24). Inset shows specific sampling points for inoculated pack studies with a fixed thermocouple (Thermocouple-K) location for each rack position.

Apple and tofu samples were sliced to approximately 0.60 cm thickness using an ethanol-sterilized knife (Oxo V-blade, Conshohocken, PA, USA), and pre-chopped kale samples (~1 cm wide strips) were used. The dehydrator unit (Weston pro-2400) was filled (24 racks) with uninoculated samples placed ~1 cm or less apart across racks, with only one product type per trial. Spot inoculated samples (*a_w_* 0.99 ± 0.01) with initial *E. coli* O157:H7 counts of 8.0 ± 0.5 log CFU/g were treated in the dehydrator at 60.0 °C (140 °F) for 24 h (apples and tofu) or 8 h (kale). Five different rack positions (rack No. 4, 8, 12, 16, and 20 from the top) were chosen to represent various height levels in the dehydration unit and inoculated samples were centered within each tray (Figure 1). During each dehydration trial, samples were pulled from each rack position and transferred to a sterile Whirl-Pak^®^ bag after 1, 2, 3, 4, and 8 h. Samples were transferred to ice bath for ~3 min, diluted with PW, stomached (Stomacher 400 circulation, VWR), and plated on to TSA. Plates were incubated for 24 ± 3 h at 35 ± 2 °C. Three independent replications were performed with an independent biological culture for each product and trial. A thin wire K-type thermocouple was inserted into the center of food samples placed at pre-determined locations (Figure 1). Wire thermocouples were attached to an 8-input data logger (OM-HL-EH-TC, Omega Engineering), with recording intervals of 10 s. For apple and tofu, *a_w_* sampling was performed every 60 min. For kale, the sampling interval was set to 30 min.

### 2.6. Isothermal Data Modeling

The primary inactivation model used was the first order kinetic, or log-linear model.

(1) $$\log\left(\frac{N}{N_{0}}\right)=-\frac{t}{D_{T}}$$

where *N* and *N*_0_ are the bacteria populations (CFU/g) at times *t* and 0, respectively, *t* (min) is isothermal treatment time, and *D_T_* is the time (min) required to reduce the bacterial population by 10-fold at a specified temperature *T* (°C).

After fitting the primary model for each set of survivor data resulting from each food matrix, temperature, and *a_w_* combination, two Bigelow-type secondary models were used to model the effect of temperature and *a_w_* on the *D*-value. The first secondary model examined only the effect of temperature for each food matrix and *a_w_* combination independently on the *D*-value:

(2) $$D_{T}\left(T\right)=D_{ref}\times 10^{\frac{T_{ref}-T}{z_{T}}}$$

where *D_ref_* is the time (min) required to achieve a 10-fold reduction at *T_ref_* (°C), the reference temperature, and *z_T_* is the temperature required to change the *D*-value by 10-fold (°C).

The second secondary model evaluated the effect of *a_w_* and temperature on the inactivation kinetics as reported in Smith et al., 2016 [22]:

(3) $$D_{T,a_{w}}\left(T,a_{w}\right)=D_{ref}\times 10^{\frac{T_{ref}-T}{z_{T}}}\times 10^{R_{aw}\times \left(a_{w}{}_{ref}-a_{w}\right)}$$

where *a_w,ref_* (unitless) was taken as a reference for this experiment, *a_w_* (unitless) is the water activity of the sample at treatment, and *R_aw_* (unitless) is the scaled impact an incremental change of *a_w_* has on the *D*-value.

Parameters for each model were estimated using a weighted ordinary least squares minimization in MATLAB (version 2019b; MathWorks, Natick, MA, USA) via the *nlinfit* function. Primary model (Equation (1)) parameters were estimated using each food matrix, temperature, and *a_w_* combination with all trials normalized then pooled. For the secondary models, parameters were estimated by incorporating Equation (2) or Equation (3) into the primary model (Equation (1)) and fitting the data globally with all experimental data combined for each food matrix. Parameter standard error (SE) and 95% confidence intervals were computed using the *nlpredci* function in MATLAB. The estimated parameter values in the text are written as the average estimate ± SE.

Model error was estimated for each predictive model with root mean squared error (RMSE; log CFU/g):

(4) $$\mathrm{RMSE}=\sqrt{\frac{\sum_{i=1}^{n}{\left[\log{\left(\frac{N}{N_{0}}\right)}_{predictied}-\log{\left(\frac{N}{N_{0}}\right)}_{observed}\right]}^{2}}{n-p}}$$

where $\log{\left(\frac{N}{N_{0}}\right)}_{predictied}$ is the predicted log reduction from the model, $\log{\left(\frac{N}{N_{0}}\right)}_{observed}$ is the experimentally obtained log reduction, *n* is the total number of observations in the dataset, and *p* is the number of model parameters.

### 2.7. Statistical Modeling

The results of isothermal experiments were examined for statistical significance by a linear model with the main effects of temperature, *a_w_*, and food matrix, as well as their two-way interactions. F-tests were used to assess statistical significance of model effects. Microbial counts were log transformed to better align with the model assumptions of normality and homogeneous variance. Pairwise comparisons for statistical significance between different food matrices were performed using the Tukey HSD method to adjust for multiple comparisons in the R-package *Emmeans* [23]. The R-studio computer program (version 1.3.959, 2009–2020 RStudio, PBC) was used for all ANOVA calculations [24].

## 3. Results and Discussion

### 3.1. Inactivation of E. coli O157:H7 during Isothermal Treatments

**Figure 2:**
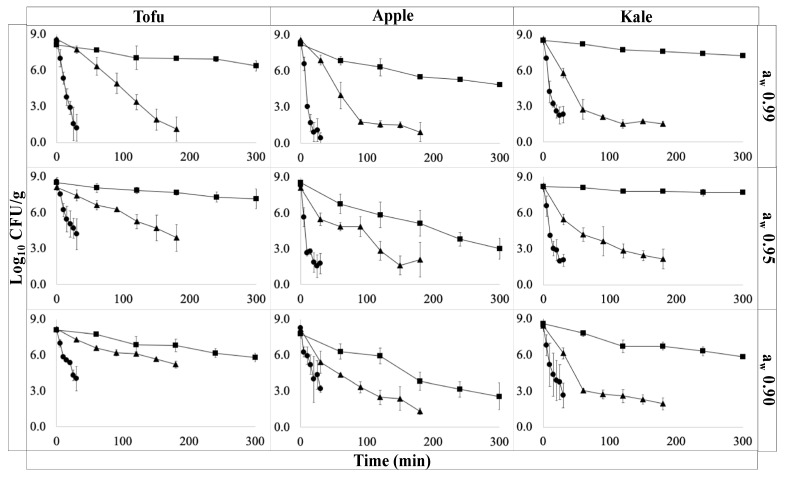
Survival plots for *E. coli* O157:H7 cocktail (log CFU/g) under different treatment temperature 49.0 °C (■), 54.5 °C (▲), and 60.0 °C (●) over time (min) during isothermal studies. Columns represent different food samples (tofu, apple, or kale) and rows represent different *a_w_* levels (0.99 ± 0.01, 0.95 ± 0.05, or 0.90 ± 0.05).

Relatively short come-up-times of 60–80 s were recorded across all *a_w_* levels. Minimal shouldering effects were observed on inactivation curves (Figure 2) particularly at higher temperatures as has been previously reported [25]. The log *D*-values were found to vary significantly by temperature and food matrix (*p* < 0.05). Pairwise comparisons of log *D*-values revealed a statistically significant difference between inactivation rates in apple and kale (*p* < 0.05). In kale, *D*_49°C_ was 86.3 min at *a_w_* level 0.90 (Table 1). Under these same conditions, the *D*-values from apple and tofu trials were 54.5 min and 107.6 min, respectively. The smaller *D*-value in apple compared to kale may be due to matrix composition factors such as lower pH (3.5) which has been shown to reduce the thermal tolerance of *E. coli* [26]. The slowed inactivation rate in tofu compared to kale may be due to the increased thickness fat, and protein content, which can confer a protective effect [27]. While the *a_w_* level had a non-statistically significant effect on inactivation, a general increase in *D*-values was observed with decreasing *a_w_* across all product types. However, the change in inactivation rates was not linearly correlated with the change in *a_w_* as has also been reported by Buerman et al., 2019 [28]. The general relationship between decreasing *a_w_* and increasing inactivation rate has been identified in a wide-range of low moisture foods, including peanut butter [29], spices, and pet food [30]. However, the specific inactivation kinetics are dependent on the target pathogen as well as the food matrix [31,32]. To date, relatively little research has been done on *E. coli* O157:H7 inactivation in low moisture foods [33,34] or in dehydration of plant-based foods [2].

The temperature-only and combined temperature/*a_w_* models are reported in Table 1. The temperature-only Bigelow-type secondary (Equation (2)) model resulted in RMSE values of 0.62 to 0.82 log CFU/g for kale, 1.12 to 1.86 log CFU/g for apples, and 1.11 to 1.31 log CFU/g in tofu. The temperature and *a_w_* model (Equation (3)) provided similar RMSE values with values of 0.75, 1.54, and 1.24 log CFU/g for kale, apple, and tofu, respectively. Notably, the model RMSE for apple models represented the worst fit among all products. Notably, the estimated *z*-value also varied the most in apple at different water activities. This could suggest that the effect of *a_w_* on the inactivation rate is temperature dependent. With temperature-only and temperature and *a_w_* models resulting in similar RMSE values, this indicates that the addition of the *a_w_* term did not lower the model’s predictive power. This is in contrast to previous work where interaction effects were not identified [35,36,37,38]. Heating rate has been previously identified as a relevant factor in inactivation models, so the combination of a moderate treatment temperatures and acidic matrixes may by unique to dehydration in apples [39,40].

### 3.2. The Impact of Sample Location on Temperature, a_w_, and Microbial Inactivation in Inoculated Pack Studies

**Figure 3:**
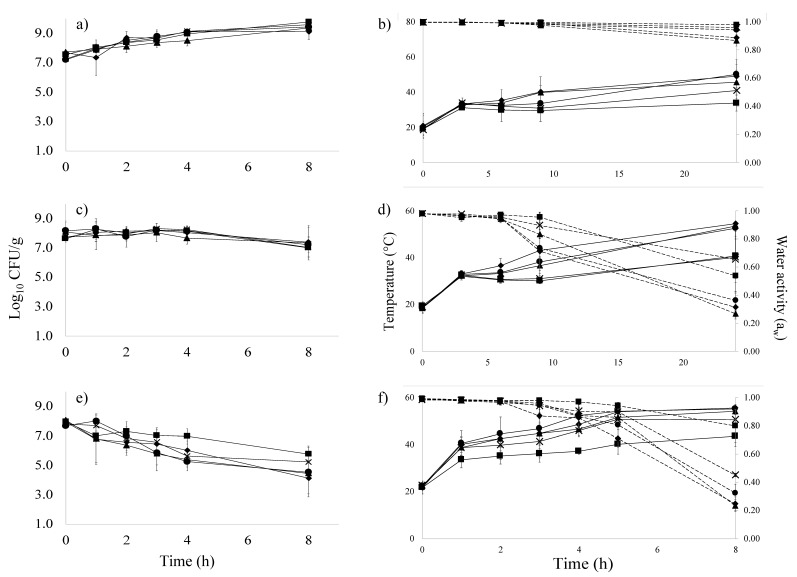
Survival plots for *E. coli* O157:H7 cocktail (log CFU/g) (Left) and simultaneous changes in sample temperature (solid lines) and *a_w_* (dashed lines) over time (Right) during dehydration of tofu (**a**,**b**), apple (**c**,**d**), and kale (**e**,**f**) at 60.0 °C. Symbols indicate sample location by rack number 4 (▲), 8 (♦), 12 (●), 16 (**X**), and 20 (■).

In dynamic dehydration studies, the temperature, *a_w_* levels, and microbiological outcomes varied by sample location within the dehydration unit (Figure 3). At the final sampling time, the average *a_w_* for apples, tofu, and kale on rack 4 were 0.27 ± 0.02, 0.89 ± 0.04, and 0.21 ± 0.05, respectively (Figure 3). The other end of the dehydrator (rack 20) at the same time, the average *a_w_* for apples, tofu, and kale were 0.54 ± 0.12, 0.98 ± 0.01, and 0.73 ± 0.17, respectively. The *a_w_* values varied significantly by rack position for apples and kale, the difference was not found to be statistically significant in tofu (*p* > 0.05).

Similarly, sliced apples taken from rack 4 achieved higher product temperatures (53.5 °C after 24 h) compared to sliced apples taken from rack 20 (40.6 °C after 24 h) (Figure 3d). In kale, a final product temperature of 54.1 °C was observed at rack 4 when compared to 43.6 °C for samples located on rack 20 at 8 h (Figure 3f). For tofu after 24 h treatment, the sample temperatures recorded at rack 4 and 20 were 45.6 °C and 33.9 °C, respectively (Figure 3b). Temperature was found to vary significantly by rack position for tofu and kale; however, the effect was not found to be statistically significant in apples (*p* > 0.05).

Microbial inactivation results were also location dependent. In kale, for example, the samples at rack 4 had a count of 4.5 ± 1.4 log CFU/g compared to 5.8 ± 0.6 log CFU/g in samples from rack 20 after 8 h (Figure 3e). For apples after 8 h, samples in the center of rack 4 had 7.4 log CFU/g survivors compared to 7.5 log CFU/g for rack 20 (Figure 3c). The difference increased after 24 h of treatment to 6.1 log CFU/g for samples on rack 4 compared to 7.3 log CFU/g survivors for samples on rack 20 (Figure S1b). Additionally, for tofu, an increase in cell count was recorded during the first 8 h of processing; however, after 24 h at 60 °C, survivor counts in tofu on rack 4 were 5.6 log CFU/g compared to 8.4 log CFU/g for samples on rack 20 (Figure S1a). While survivor counts were numerically different based on rack position, the effect of rack position on thermal inactivation of *E. coli* O157:H7 in inoculated pack studies was not statistically significant after 8 h of treatment across all products (Figure 3).

Overall, locations with the highest temperature also had the lowest *a_w_* and greatest microbial inactivation suggesting that, under the range of treatments evaluated in this process, product temperature was indicative of microbial inactivation despite its associated impact on moisture removal. Samples taken from rack 4 had the highest recorded temperature, lowest *a_w_*, and fewest survivors followed by rack 8, 12, 16, then 20 which had the lowest temperature, highest *a_w_*, and most survivors across all three food types. Rack 4 was the most distant from the heating source but is at the top of the dehydration unit (Figure 1). Various unit design features (location of heating source, air circulation system) as well as operational factors (fill density, tray position) have also been shown to impact heat distribution and *a_w_* dynamics during dehydration [12,13]. The extent to which mediating variables impact heat distribution may render *a priori* identification of the cold spot difficult. Under the conditions evaluated in this study, the “cold spot” represented the worst-case-scenario, despite the more rapid decrease in *a_w_* in warmer locations [3].

### 3.3. Changes in E. coli O157:H7 during Inoculated Pack Studies

Throughout the duration of the 24 h dehydration process, sample temperatures did not achieve the set temperature of 60 °C (Figure 3b,d,f), resulting in conditions of temperature abuse [15,16]. These conditions supported the initial outgrowth of *E. coli* O157:H7 in tofu. During the first 8 h of tofu dehydration, the *E. coli* O157:H7 count increased by ~1 log CFU/g resulting in a total count of ~9.5 log CFU/g (Figure 3a). By the 24 h sampling point, a decline in cell count was observed (final count 7.2 ± 1.7 log CFU/g after 24 h).

By contrast to tofu experiments, the *E. coli* O157:H7 counts in apples (Figure 3c) were reduced by 0.67 log CFU/g under the same treatment conditions due to the inhibitory effect of the matrix pH (3.5), more rapid increase in temperature, and decrease in *a_w_*. The *a_w_* of apple slices was reduced below 0.90 after ~8 h of treatment, and an average final *a_w_* of 0.35 was recorded after 24 h of treatment (Figure 3d). For kale, *E. coli* O157:H7 counts were reduced by 3.1 log CFU/g within 8 h of processing time as the *a_w_* level reached ~0.85 (the cut-off for pathogenic bacterial growth) within the first 5 h of treatment. An average final *a_w_* for kale of 0.39 was recorded after 8 h of treatment (Figure 3f) as a consequence of greater heat penetration compared to tofu slices (Figure 3e). Inoculated pack data suggested that the treatments necessary to achieve the final *a_w_* used for shelf-stability would not simultaneously provide a 5 log CFU/g *E. coli* O157:H7 reduction. In fact, minimal overall pathogen reduction was achieved in apple or tofu. The use of an acid bath or brine prior to dehydration has been suggested as a means to decrease the initial microbial load and increase cumulative microbial inactivation in dehydration by ~1 log CFU/g [5,6,7]. Additional operational changes that increase heat distribution may also enhance inactivation rates.

Previous studies on the inactivation of *E. coli* in apple slices during dehydration at 62.8 °C for 6 h achieved a reduction of 3.5 log CFU/g [5] and 3.1 log CFU/g [6]. These studies utilized a higher operational temperature (62.8 °C rather than 60 °C) and only included the test samples (*n* = 6 to 10) alone in the entire dehydrator unit. By contrast, the dehydration unit in this study was completely filled with product (*n* = 575 to 1750 pieces) to better align with commercial practices. High fill density may have impeded thermal distribution. Indeed, sample product temperatures never achieved the set operational temperatures even after prolonged periods of time (Figure 3b,d,f). Dynamic processing simulations which do not account for the impact of fill density on microbial inactivation may overestimate predicted lethality. The complexity of developing accurate models for both isothermal [41,42] and dynamic processes [43] has been identified as relevant in validation, even as there has been increased recognition of the food safety challenges in low *a_w_* foods which cannot simply be controlled by preventing contamination [44,45]. The research presented here contributes to the ongoing effort to improve thermal inactivation stratagies for the growing sector of minimally processed, plant-based foods.

## 4. Conclusions

Dehydration of plant-based foods at moderate temperatures (≤60 °C) is representative of growing interest in minimal processing. However, depending on operational conditions, the treatments applied to achieve shelf-stability may not provide sufficient inactivation in the pathogen of concern. Minimal microbial reductions, and in the case of tofu, initial growth, were achieved under dynamic drying conditions at 60 °C, indicating that moderate temperature dehydration under the conditions assessed here does not provide an effective thermal inactivation treatment. Although the isothermal data suggested that significant *E. coli* O157:H7 inactivation could be achieved, that model is only appropriately applied when the temperature is between 49 and 60 °C and when the *a_w_* is ≥ 0.90. Under dynamic dehydration treatments at 60 °C, these two conditions were never achieved simultaneously. Throughout the inoculated pack experiment, the product conditions either: (i) potentially supported microbial growth (<45 °C and *a_w_* > 0.90) or (ii) were too dry to apply the isothermal inactivation model (*a_w_* < 0.90). While there have been many recent studies using isothermal data to construct models to estimate dynamic microbial inactivation in various low *a_w_* food systems, this approach would not be appropriate under the conditions of moderate temperature dehydration of plant-based foods tested here. Given the minimal lethality, additional treatments such as an initial acid wash, equipment modification to enhance thermal distribution and penetration, or an increase in the processing temperature may be necessary if a 5 log CFU/g reduction is targeted. Variation among sites within the dehydration unit and the limitations of existing modeling strategies indicate that in-unit testing may be necessary to identify critical process control parameters and validate a heat-assisted dehydration kill step for microbial food safety in plant-based food products.

## Figures and Tables

**Table 1:** Thermal inactivation kinetics for *E. coli* O157:H7 in apples, tofu, and kale determined using modified Bigelow-type models based on (Equation (2)) temperature or (Equation (3)) temperature and *a_w_* during isothermal studies.

Product	Estimations from Equation (2) (Temperature Only)					Estimations from Equation (3) (Temperature & *a_w_*)			
	*a_w_*	Temp (°C)	*D*-Value (min ± SE)	*z*-Value (°C ± SE)	RMSE (log CFU/g)	*D*_49°C_ (min ± SE)	*z*-Value (°C ± SE)	*R_aw_* (Unitless ± SE)	RMSE (log CFU/g)
Apple	0.99	49	78.2 ± 8.2	7.4 ± 0.6	1.26	71.9 ± 10.1	8.7 ± 0.5	1.1 ± 0.7	1.54
		54.5	19.3 ± 2.3						
		60	3.0 ± 0.5						
	0.95	49	45.9 ± 3.9	8.9 ± 1.1	1.86				
		54.5	25.2 ± 2.9						
		60	3.5 ± 0.5						
	0.9	49	54.5 ± 6.8	10.9 ± 1.2	1.12				
		54.5	23.1 ± 2.5						
		60	5.5 ± 0.8						
Tofu	0.99	49	173.3 ± 35.1	6.9 ± 0.3	1.31	107.3 ± 14.2	7.7 ± 0.4	0.6 ± 0.5	1.24
		54.5	24.1 ± 1.6						
		60	3.8 ± 0.3						
	0.95	49	173.5 ± 16.6	6.9 ± 0.4	1.11				
		54.5	43.3 ± 4.5						
		60	6.1 ± 0.8						
	0.9	49	107.6 ± 12.7	7.6 ± 0.8	1.18				
		54.5	55.4 ± 4.6						
		60	6.7 ± 0.6						
Kale	0.99	49	174.1 ± 27.2	7.5 ± 0.6	0.62	143.9 ± 17.0	7.0 ± 0.3	3.3 ± 0.5	0.75
		54.5	19.6 ± 2.8						
		60	3.8 ± 0.4						
	0.95	49	257.4 ± 71.0	7.1 ± 0.5	0.64				
		54.5	22.8 ± 3.0						
		60	4.0 ± 0.4						
	0.9	49	86.3 ± 10.8	8.7 ± 0.8	0.82				
		54.5	22.2 ± 3.1						
		60	4.8 ± 0.8						

## Data Availability

The data presented in this study are openly available through the following citation: Yadwinder Singh Rana, Philip M. Eberly, Quincy J. Suehr, Ian M. Hildebrandt, Bradley P. Marks, and Abigail B. Snyder. (2021) Data from: Survival of Escherichia coli O157:H7 during moderate temperature dehydration of plant-based foods. [Dataset] Cornell University eCommons Repository. https://doi.org/10.7298/tn9b-gr76.

## Supplementary Materials

The following are available online at https://www.mdpi.com/article/10.3390/foods10092162/s1, Figure S1: Survival plots for *E. coli* O157:H7 cocktail (average Log CFU/g) in tofu (a) and apples (b) when treated at 60 °C for up to 24 h during inoculated pack studies at two different tray locations.

## References

[1] Beuchat L.R., Komitopoulou E., Beckers H., Betts R.P., Bourdichon F., Fanning S., Joosten H.M., Ter-Kuile B.H. (2013). Low-water activity foods: Increased concern as vehicles of foodborne pathogens. J. Food Prot..

[2] Bourdoux S., Li D., Rajkovic A., Devlieghere F., Uyttendaele M. (2016). Performance of drying technologies to ensure microbial safety of dried fruits and vegetables. Comp. Rev. Food Sci. Food Saf..

[3] Jayaraman K.S., Das-Gupta D.K., Mujumdar A.S. (2014). Drying of fruits and vegetables. Handbook of Industrial Drying.

[4] Saravacos G.D., Kostaropoulos A.E., Saravacos G.D., Kostaropoulos A.E. (2002). Food dehydration equipment. Handbook of Food Processing Equipment.

[5] Burnham J.A., Kendall P.A., Sofos J.N. (2001). Ascorbic acid enhances destruction of *Escherichia coli* O157:H7 during home-type drying of apple slices. J. Food Prot..

[6] Derrickson-Tharrington E., Kendall P.A., Sofos J.N. (2005). Inactivation of *Escherichia coli* O157:H7 during storage or drying of apple slices pretreated with acidic solutions. Int. J. Food Microbiol..

[7] Dipersio P.A., Kendall P.A., Calicioglu M., Sofos J.N. (2003). Inactivation of *Salmonella* during drying and storage of apple slices treated with acidic or sodium metabisulfite solutions. J. Food Prot..

[8] Centers for Disease Control and Prevention (CDC) (2020). List of Selected Multistate Foodborne Outbreak Investigations. https://www.cdc.gov/foodsafety/outbreaks/multistate-outbreaks/outbreaks-list.html.

[9] European Food Safety Authority (EFSA), Panel on Biological Hazards (BIOHAZ) (2013). Scientific Opinion on the Risk Posed by Pathogens in Food of Non-Animal Origin. Part 1 (Outbreak Data Analysis and Risk Ranking of Food/Pathogen Combinations). EFSA J..

[10] Centers for Disease Control and Prevention (CDC) (2018). Multistate Outbreak of *Salmonella* Typhimurium Infections Linked to Dried Coconut (Final Update). https://www.cdc.gov/salmonella/typhimurium-03-18/index.html.

[11] Centers for Disease Control and Prevention (CDC) (2019). Outbreak of E. coli Infections Linked to Flour (Final Update). https://www.cdc.gov/ecoli/2019/flour-05-19/index.html.

[12] Mujumdar A.S., Mujumdar A.S. (2014). Principles, classification, selection of dryers. Handbook of Industrial Drying.

[13] Kiang C.S., Jon C.K., Mujumdar A.S. (2014). Heat Pump Drying Systems. Handbook of Industrial Drying.

[14] Cai S., Worobo R.W., Snyder A.B. (2019). Combined effect of storage condition, surface integrity, and length of shelf life on the growth of *Listeria monocytogenes* and spoilage microbiota on refrigerated ready-to-eat products. J. Food Prot..

[15] Lampel K., Al-Khaldi S., Cahill S., Food and Drug Administration (FDA) (2012). Bad Bug Book, Foodborne Pathogenic Microorganisms and Natural Toxins.

[16] Linton R. (2003). Food Safety Hazards in Foodservice and Food Retail Establishments. http://citeseerx.ist.psu.edu/viewdoc/download?doi=10.1.1.222.2542&rep=rep1&type=pdf.

[17] Food and Drug Administration (FDA) (2011). H.R. 2751 FDA Food Safety Modernization Act. https://www.fda.gov/food/guidance-regulation-food-and-dietary-supplements/food-safety-modernization-act-fsma.

[18] Santillana-Farakos S.M., Frank J.F., Schaffner D.W. (2013). Modeling the influence of temperature, water activity and water mobility on the persistence of *Salmonella* in low-moisture foods. Int. J. Food Microbiol..

[19] Valdramidis V.P., Geeraerd A.H., Gaze J.E., Kondjoyan A., Boyd A.R., Shaw H.L., Van Impe J.F. (2006). Quantitative description of *Listeria monocytogenes* inactivation kinetics with temperature and water activity as the influencing factors; model prediction and methodological validation on dynamic data. J. Food Eng..

[20] Villa-Rojas R., Tang J.M., Wang S.J., Gao M.X., Kang D.H., Mah J.H., Gray P., Sosa-Morales M.E., Lopez-Malo A. (2013). Thermal inactivation of *Salmonella Enteritidis* PT 30 in almond kernels as influenced by water activity. J. Food Prot..

[21] Snyder A.B., Perry J.J., Yousef A.E. (2016). Developing and optimizing bacteriophage treatment to control enterohemorrhagic *Escherichia coli* on fresh produce. Int. J. Food Microbiol..

[22] Smith D.F., Hilderbrandt I.M., Casulli K.E., Dolan K.D., Marks B.P. (2016). Modeling the effect of temperature and water activity on the thermal resistance of *Salmonella* Enteritidis PT 30 in wheat flour. J. Food Prot..

[23] Lenth R. (2020). Emmeans: Estimated Marginal Means, aka Least-Squares Means. R Package Version 1.4.8. https://CRAN.R-project.org/package=emmeans.

[24] R Core Team (2020). R: A Language and Environment for Statistical Computing.

[25] Mattick K.L., Jorgensen F., Wang P., Pound J., Vandeven M.H., Ward L.R., Legan J.D., Lappin-Scott H.M., Humphrey T.J. (2001). Effect of challenge temperature and solute type on heat tolerance of *Salmonella* serovars at low water activity. Appl. Environ. Microbiol..

[26] Usaga J., Worobo R.W., Padilla-Zakour O.I. (2014). Effect of acid adaptation and acid shock on thermal tolerance and survival of *Escherichia coli* O157:H7 and O111 in apple juice. J. Food Prot..

[27] Amaha M., Sakaguchi K.I. (1954). Effects of carbohydrates, proteins, and bacterial cells in the heating media on the heat resistance of *Clostridium sporogenes*. J. Bacteriol..

[28] Buerman E.C., Worobo R.W., Padilla-Zakour O.I. (2019). Thermal resistance of xerophilic fungi in Low-Water-Activity (0.70 to 0.80) confectionery model foods. J. Food Protect..

[29] He Y.S., Li Y., Salazar J.K., Yang J.Y., Tortorello M.L., Zhang W. (2013). Increased water activity reduces the thermal resistance of *Salmonella enterica* in peanut butter. Appl. Environ. Microbiol..

[30] Gautam B., Govindan B.N., Gänzle M., Roopesh M.S. (2020). Influence of water activity on the heat resistance of *Salmonella enterica* in selected low-moisture foods. Int. J. Food Microbiol..

[31] Chen L., Wei X., Chaves B.D., Jones D., Ponder M.A., Subbiah J. (2021). Inactivation of *Salmonella* enterica and *Enterococcus faecium* NRRL B2354 on cumin seeds using gaseous ethylene oxide. Food Microbiol..

[32] Bianchini A., Stratton J., Weier S., Hartter T., Plattner B., Rokey G., Hertzel G., Gompa L., Martinez B., Eskridge K.M. (2014). Use of Enterococcus faecium as a surrogate for Salmonella enterica during extrusion of a balanced carbohydrate-protein meal. J. Food Protect..

[33] Zhang S., Zhang L., Cheng T., Guan X., Wang S. (2020). Effects of water activity, temperature and particle size on thermal inactivation of *Escherichia coli* ATCC 25922 in red pepper powder. Food Control.

[34] Brar P.K., Proano L.G., Friedrich L.M., Harris L.J., Danyluk M.D. (2015). Survival of *Salmonella, Escherichia coli* O157:H7, and *Listeria monocytogenes* on raw peanut and pecan kernels stored at −24, 4, and 22 °C. J. Food Protect..

[35] Jin Y., Pickens S.R., Hildebrandt I.M., Burbick S.J., Grasso-Kelley E.M., Keller S.E., Anderson N.M. (2018). Thermal inactivation of *Salmonella* Agona in Low–Water Activity foods: Predictive models for the combined effect of temperature, water activity, and food component. J. Food Protect..

[36] Jin Y., Tang J., Zhu M. (2020). Water activity influence on the thermal resistance of *Salmonella* in soy protein powder at elevated temperatures. Food Control.

[37] Liu S., Rojas R.V., Gray P., Zhu M., Tang J. (2018). *Enterococcus faecium* as a *Salmonella* surrogate in the thermal processing of wheat flour: Influence of water activity at high temperatures. Food Microbiol..

[38] Wei X., Lau S.K., Chaves B.D., Danao M.C., Agarwal S., Subbiah J. (2020). Effect of water activity on the thermal inactivation kinetics of *Salmonella* in milk powders. J. Dairy Sci..

[39] Zhang S., Zhang L., Lan R., Zhou X., Kou X., Wang S. (2018). Thermal inactivation of *Aspergillus flavus* in peanut kernels as influenced by temperature, water activity and heating rate. Food Microbiol..

[40] Xu J., Tang J., Jin Y., Song J., Yang R., Sablani S.S., Zhu M. (2018). High temperature water activity as a key factor influencing survival of *Salmonella* Enteritidis PT30 in thermal processing. Food Control.

[41] Giannakourou M.C., Stoforos N.G. (2017). A theoretical analysis for assessing the variability of secondary model thermal inactivation kinetic parameters. Foods.

[42] Lang E., Chemlal L., Molin P., Guyot S., Alvarez-Martin P., Perrier-Cornet J.M., Dantigny P., Gervais P. (2017). Modeling the heat inactivation of foodborne pathogens in milk powder: High relevance of the substrate water activity. Food Res. Int..

[43] Brackett R.E., Ocasio W., Waters K., Barach J., Wan J. (2014). Validation and Verification: A practical, industry-driven framework developed to support the requirements of the Food Safety Modernization Act (FSMA) of 2011. Food Protect. Trends.

[44] Brar P.K., Danyluk M.D. (2018). Nuts and grains: Microbiology and preharvest contamination risks. Preharvest Food Saf..

[45] Cai S., Phinney D., Heldman D., Snyder A.B. (2020). All treatment parameters affect environmental surface sanitation efficacy, but their relative importance depends on the microbial target. Appl. Environ. Microbiol..

