# Supplementary material for: Survival of Escherichia coli O157:H7 during Moderate Temperature Dehydration of Plant-Based Foods
Source: Foods. 2021 Sep 13;10(9):2162. doi: 10.3390/foods10092162 (PMC8469793; doi:10.3390/foods10092162)
Supplement: Supplementary file 1 [file foods-10-02162-s001.zip › foods-1346503-SI.pdf]

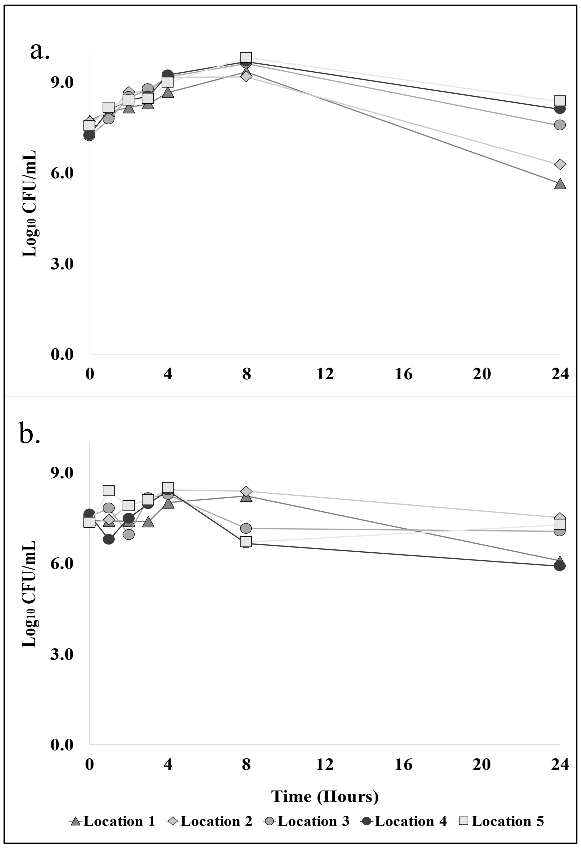

Figyre S1: Survival plots for *E. coli* O157:H7 cocktail (average Log CFU/g) in tofu (a) and apples (b) when treated at 60°C for up to 24 hours during inoculated pack studies at two different tray locations.
